# Investigating the Impact of Coffee Pulp on Milk Yield and Blood Parameters in Awassi Ewes

**DOI:** 10.3390/ani16182879

**Published:** 2026-09-13

**Authors:** Belal S. Obeidat, Hadil S. Subih, Linda Alyahya

**Affiliations:** 1 Department of Animal Production, Faculty of Agriculture, Jordan University of Science and Technology, Irbid 22110, Jordan; 2 Department of Nutrition and Food Technology, Faculty of Agriculture, Jordan University of Science and Technology, P.O. Box 3030, Irbid 22110, Jordan; 3 School of Pharmaceutical Sciences, Universiti Sains Malaysia, Gelugor 11800, Pulau Pinang, Malaysia

**Keywords:** Awassi ewes, coffee pulp, nursing performance, production cost, blood traits

## Abstract

Using industrial waste in livestock feed is crucial for lowering production costs while reducing the environmental impact. Accordingly, the goal of this study was to evaluate the effects of feeding coffee pulp (COP) on the Awassi ewes’ milk production, nutrient digestibility, and blood parameters. Thirty 5-year-old Awassi ewes with an initial body weight of 56.7 ± 3.72 kg were selected for the experiment. Three dietary rations were used, and ten ewes were randomly assigned to each diet for nine weeks, including one week of adaptation. The diets were: (1) 0 COP (CON), (2) 100 COP (COP100), and (3) 200 g/kg COP of dietary dry matter (COP200). Coffee pulp was included at 100 and 200 g/kg of dietary DM; as a result, barley grain increased whereas soybean meal and wheat straw decreased as COP inclusion increased, while the diets remained approximately isonitrogenous. We evaluated effects on intake, performance, milk production and composition, digestibility, nitrogen balance, and selected serum biochemical parameters. Nutrient intake did not differ among dietary groups (*p* > 0.05). Milk lactose concentration was significantly higher in COP100 and COP200 than in CON (*p* = 0.015). No adverse changes were detected in the measured productive responses or selected serum biochemical parameters. The cost of milk production decreased with coffee pulp inclusion, from approximately US$ 0.359/kg milk in CON to US$0.325 and US$ 0.300/kg milk in COP100 and COP200, respectively (*p* = 0.002), corresponding to reductions of approximately 9.5% and 16.4%. Blood urea nitrogen was higher (*p* = 0.017) in ewes that consumed the COP diets than in those that consumed the CON diet. These results indicated that substituting conventional feedstuffs in ewes’ diets with up to 200 g/kg of COP was effective and reduced milk production costs.

## Simple Summary

Coffee pulp is an agro-industrial by-product that may provide a locally available alternative feed resource for ruminants while valorizing processing residues and reducing reliance on conventional feed ingredients. This research evaluated the use of coffee pulp in the diet of lactating ewes for its effects on milk production and composition, nutrient intake and digestibility, lamb growth, nitrogen balance, and selected serum biochemical parameters. Coffee pulp inclusion did not affect the performance of ewes and their lambs. However, results showed that milk production cost was lower with coffee pulp inclusion.

## 1. Introduction

Various feed industry by-products are being explored as replacements for conventional feedstuffs, as demand for more affordable feed that is easy for animals to consume increases profitability and has no detrimental impact on their productivity or health [1]. Ruminants can effectively utilize many fibrous feed resources through ruminal microbial fermentation; however, the extent of fiber digestion varies according to feed composition, processing, and the characteristics of the fiber fraction. This capability enables them to use agro-industrial by-products, which typically contain substantial amounts of fiber, transforming fiber-dense materials into essential nutrients and playing a crucial role in the effective use of agricultural and industrial waste. Coffee pulp (COP) is an Agro-industrial waste that has recently been used as an alternative feed for ruminants [2,3]. Coffee generates a significant amount of waste during its processing. Such waste consists of COP, which makes up approximately 500–600 g/kg of the entire harvest [2]. In this study, COP refers specifically to the coffee-processing residue used as the dietary ingredient; coffee husk, mucilage, spent coffee grounds, and other coffee residues are discussed separately in previous studies.

Feeding COP to ruminants might help reduce pollution from coffee processing. Yet, due to its moderate levels of caffeine, free phenols, and tannins, all of which are known to be toxic in high concentrations to numerous biological processes, including the rumen microbiota, the widespread use and management of COP as animal feedstuff worldwide continues to be a challenge [4,5]. The purpose of processing coffee fruit is to produce a range of by-products, minimize process losses, and produce dry pulp coffee without affecting its quality. The procedure starts with gathering ripe fruits, then crushing and categorizing them, followed by removing the mucilage and drying the grain. At this point, by-products such as pulp, husk, and mucilage are produced. The husk and mucilage make up 560 g/kg of coffee grain [6]. About 436 g/kg of the fresh fruit is made up of the shell, often known as the pulp. Studies reported that partial replacement of traditional feed with COP ranging from 200 to 400 g/kg had no adverse effect on animal performance [7].

Results from several studies on feeding ruminants COP have been inconsistent. The feed conversion ratio (FCR), weight gain, and feed consumption were unaffected when lambs were given up to 280 g/kg COP [8]. Similarly, Salinas-Rios et al. [9] reported that lambs’ performance was unaffected by adding up to 160 g/kg COP with 50 g/kg molasses to their diets. In addition, Obeidat et al. [1] found that including COP in the diets of growing lambs at 50 and 100 g/kg DM produced similar growth performance across treatments while reducing production costs in the COP diets compared with the control diet. The effect of offering coffee by-products on milk production and on hematological traits was investigated in goats, cows, and ewes [10,11,12]. Others reported that feeding coffee by-products had no effect on dairy cows’ milk yield or composition [13]. Although coffee pulp has been evaluated in growing sheep and other ruminants, information remains limited on its use at 100 and 200 g/kg DM in lactating Awassi ewes, particularly regarding milk production and composition, nutrient digestibility, nitrogen balance, and selected serum biochemical parameters. Accordingly, we studied the effects of COP inclusion in the diets of lactating Awassi ewes on nursing performance. We hypothesized that including COP at 100 or 200 g/kg DM would maintain lactational performance and nutrient utilization while reducing calculated feed cost without adversely altering the measured serum biochemical parameters.

## 2. Materials and Methods

The study was conducted at the Animal Field facility of Jordan University of Science and Technology. The Institutional Animal Care and Use Committee of Jordan University of Science and Technology approved all study procedures (16/04/12/216). Following estrous synchronization, thirty 5-year-old Awassi ewes nursing a single lamb were selected from the synchronized group and randomly allocated to the three dietary treatments. Thirty 5-year-old Awassi ewes were selected, with initial body weights of 56.7 ± 3.72 kg. Ten ewes were randomly assigned to each of the three dietary rations. Only ewes nursing a single male lamb (1–2 days of age) were selected. The diets were: (1) 0 COP (CON), (2) 100 COP (COP100), and (3) 200 g/kg COP of dietary DM (COP200). Coffee pulp was included at 100 and 200 g/kg of dietary DM; as shown in Table 1, barley grain increased while soybean meal and wheat straw decreased with increasing COP inclusion. The COP was acquired from Al-Hassan Industrial City in Irbid, Jordan. The ewes were housed individually in separate shaded enclosures (0.75 × 1.5 m) and fed their respective isonitrogenous diets alongside their lambs. Experimental diets were offered once daily at 08:00, and daily feed intake was determined from feed offered and refusals. The experimental diets were offered in a manner that prevented direct access by lambs.

The first week was used as an adaptation period to allow the ewes to acclimate to the experimental diets and housing conditions, followed by an eight-week data-collection period. The trial lasted roughly nine weeks. Ewes received the experimental diets once daily at 8:00 a.m. throughout the trial, and they had free access to water. The diets were suitable for nursing ewes, with a crude protein (CP) content of 160 g/kg ([14]; Table 1). To minimize feed restriction, feed was offered in excess of expected daily intake. Feed was offered in excess of expected daily intake (approximately 110% of the preceding intake) to minimize feed restriction, and the amount offered was adjusted according to individual intake and refusals. Feed intake was measured by weighing the daily feed amount and recording feed refusals. Body weight was recorded at two-week intervals under consistent conditions, at 08:00 before the daily feed allowance was offered, using the same calibrated scale throughout the experiment.

For milk-yield estimation, lambs were separated from their dams for 3 h, after which the ewes were manually milked. Oxytocin was administered intravenously (0.75 mL) to facilitate milk ejection, followed by a second milking. The milk collected during the standardized procedure was used to estimate milk yield. Daily milk yield was estimated by multiplying the milk obtained during the standardized 3-h separation/milking interval by eight, following the established procedure used for suckling ewes [15]. This provides an estimate of 24-h milk production rather than a direct 24-h collection. Milk samples (125 mL/ewe) were analyzed for total solids, crude protein (CP), and fat. Total solids were determined by oven drying at 55 °C. CP was determined by the Kjeldahl method (N = 6.38), and fat was determined by the Gerber method (Gerber Instruments, K. Schnider and Co., Effretikon, Switzerland). As stated by Baldi et al. [16], the formula used to determine the energy value of milk (MEV; Kcal/kg) was 203.8 + (8.36 × fat%) + (6.29 × protein%). Energy-corrected milk (ECM; kg/d) was calculated as 0.3246 × milk yield + (12.86 × fat yield) + (7.04 × protein yield).

Chemical analysis was performed on diet samples and feed refusals. The DM was determined by drying the samples overnight at 105 °C in a forced-air oven. The CP content was determined using the Kjeldahl procedure (N = 6.25). We used an ANKOM2000 fiber analyzer (ANKOM Technology Corporation, Macedon, NY, USA) to determine neutral detergent fiber (NDF) and acid detergent fiber (ADF). Feed refusal was subtracted from the amount of feed given to determine nutrient consumption.

At the end of the lactation trial, 5 ewes from each treatment (15 ewes in total) were randomly selected for a separate 10-day metabolic trial to determine nutrient digestibility and nitrogen (N) balance. Ewes were fed the same diets. The first five days of the 10-day trial were allocated to adaptation. During the five-day collection period, feces and urine were quantitatively collected over each 24 h period. Daily fecal and urine outputs were recorded for each ewe, and representative subsamples were retained for laboratory analysis. Plastic troughs in the cages provided the ewes with unrestricted access to food and water. After fecal samples were collected and weighed, 10% of each sample was retained for additional examination to determine the digestibility of DM, CP, NDF, and ADF.

To determine the N balance, urine samples were collected in plastic containers, weighed, and 5 g/100 g of each sample was stored at −20 °C for N analysis. To reduce ammonia losses, 50 mL of 6 N HCl was added to each container. Each ewe’s urine and feces samples were mixed at the end of the study. Fecal samples were dried in a forced-air oven at 55 °C for a few days before being weighed to determine DM concentration. After being ground up in a Wiley mill (Brabender OHG, Duisburg, Germany) to pass through a 1 mm screen, following AOAC procedures [17], the feed and fecal samples were put in plastic bags at room temperature in anticipation of further testing. The DM, CP, NDF, and ADF were determined in diets and fecal samples. Urine samples were collected to assess nitrogen (N) balance. Each ewe’s samples were assembled, and the N concentration was determined using the Kjeldahl procedure. The ewes’ N intake was calculated by multiplying DM intake by the N content of each diet. The amount of N lost in the urine and feces was calculated by multiplying the N content by the outputs related to it. N retained (g/d) was calculated as N intake − fecal N − urinary N. N retention (%) was calculated as (N retained/N intake) × 100.

Before feeding, each ewe had three blood samples drawn at the beginning, middle, and end of the experiment. Samples were collected from the jugular vein at 8 a.m. using standard Vacutainers and then centrifuged for 15 min at 3000 rpm. Serum samples were stored at −20 °C until the day of testing after instant separation. A spectrophotometer (JENWAY 6105 UV/Vis, Model 6105, Jeneway LTD Felsted, Dunmow ESSEX CM6 3 LB, UK) and commercial kits (BioSystems, S. A., Barcelona, Spain) were used in accordance with the manufacturer’s instructions to measure the serum concentrations of creatinine, alkaline phosphatase (ALK-P), alanine aminotransferase (ALT), aspartate aminotransferase (AST), glucose, and blood urea N.

### Statistical Analysis

The statistical data were analyzed using a completely randomized design in SAS MIXED [18]. Animals were included as a random effect. Repeated measurements of serum blood contents and milk production were analyzed using a mixed model including treatment, time, and treatment × time as fixed effects and ewe as the experimental subject/random effect. The covariance structure was selected using an information criterion. Treatment × time interactions were retained and reported irrespective of significance. For each analysis, the appropriate covariance structure was selected from the compound symmetric, autoregressive order one, and unstructured structures (based on the Schwarz Bayesian criterion). The statistical model was applied separately to repeated and single-measure outcomes as appropriate. Other data, such as nutrient intake, digestibility and N balance, were analyzed using dietary treatment as the fixed effect and ewe as the experimental unit. A significant difference was defined as *p* < 0.05.

## 3. Results

Table 1 presents the chemical composition of the diets offered to Awassi ewes. Coffee pulp that was added to the different diets had a greater amount of NDF and ADF (517 and 339 g/kg on a DM basis, respectively), while CP was 168 g/kg on a DM basis. The NDF and ADF concentrations of the experimental diets were 297, 284, and 309 g/kg DM and 138, 136, and 141 g/kg DM for COP100, COP200, and CON, respectively. All experimental diets were formulated to be iso-nitrogenous with 160 g/kg CP of dietary DM. Feed cost (US$/ton) was calculated by summing the cost contribution of each ingredient according to its inclusion rate and 2026 purchase price. Feed cost decreased from US$ 426/ton in CON to US$ 380/ton in COP100 and US$ 334/ton in COP200, corresponding to reductions of 10.8% and 21.6%, respectively.

Table 2 demonstrates the nutrient intake of ewes offered the experimental rations. Dietary treatments did not differ significantly in DM, CP, NDF, or ADF intake (*p* > 0.05). Ewe BW and lamb growth were also not significantly affected by dietary treatment (Table 3).

Table 4 shows the effect of feeding COP to Awassi ewes on milk yield and composition. There were no treatment × period differences in milk yield (*p* = 0.8645) or milk composition (*p* ≥ 0.1287). Milk lactose concentration was significantly affected by dietary treatment (*p* = 0.015), whereas milk yield and the other measured milk components were not (*p* > 0.05). Coffee pulp inclusion reduced milk-production cost (*p* = 0.002), corresponding to reductions of approximately 9.5% and 16.4% relative to CON.

Nutrient digestibility and N balance, measured in the separate metabolic trial using five ewes per treatment, were not significantly affected by dietary treatment (Table 5; *p* > 0.05). Table 6 presents the effects of feeding COP on selected serum biochemical parameters. There were no treatment × period differences in serum metabolites (*p* ≥ 0.1158) among treatments. Blood urea nitrogen (BUN) was higher in ewes receiving the COP diets than in CON (*p* = 0.017). Other blood metabolites such as glucose, cholesterol, HDL, LDL, triglycerides, creatinine, AST, ALT, and ALP did not change with the addition of COP to the diets (*p* > 0.05).

## 4. Discussion

Coffee pulp chemical composition was similar to other studies, although NDF and ADF contents were greater in the current study [19]. Variations in content may be due to the method used to process the coffee cherry. Estrada-Flores et al. [20] observed that sun-drying increased the NDF content of the coffee pulp used in their experiment. Feed cost was reduced by the addition of COP, which reflects the economic benefits of using coffee pulp in ruminants’ feed. Awawdeh et al. [21] reported that substituting conventional feedstuffs in sheep diets with different levels of alternative feed was effective and reduced the cost of production without triggering health problems in small ruminants. The calculations indicate that COP inclusion reduced the estimated feed cost of the diets and the cost per kilogram of milk under the conditions of this experiment.

Nutrient intake was not altered when ewes consumed COP-supplemented diets. Other studies reported similar results, although some noted increases in DM intake [20] and EE intake [18]. These findings suggest that including COP did not measurably affect diet palatability, as feed intake remained similar among treatments. As reported by other studies, adding coffee by-products to diets at different levels showed no detrimental effects on the nutrient intake of lambs [8], the total nitrogen intake of dairy heifers [22], or the DM intake of sheep [23].

Ewes’ body weight and the growth rate of their lambs were not influenced by the addition of COP. Carta et al. [12] noticed similar results when they used coffee by-products in dairy goats’ ration. Including coffee by-products in ewes’ rations was reported to have no effect on weight gain, body condition score, or DM intake [10]. As described by other researchers, adding coffee by-products to rations reduced ewes’ feed intake because some components may reduce palatability, such as alkaloids and polyphenols [24]. Under the inclusion levels tested in the present study, COP did not significantly alter feed intake, suggesting that the inclusion levels did not measurably impair intake under the experimental conditions.

Milk production and composition were not altered by COP inclusion, except for milk lactose content, which increased with increasing COP in ewes’ diets. Opposite results were reported by others; an increase in milk yield was noticed with the inclusion of coffee by-products in dairy ewes [10], and decreased milk yield was observed in dairy goats that consumed coffee residues [12]. Similar to our findings, dairy cows that consumed coffee pulp had no significant improvement in milk production, although an increase in lactose content was noticed [7], milk yield was numerically greater for cows that consumed COP. Researchers attributed this result to increased nutrient intake, specifically non-fibrous sugars, among cows that consumed the COP-based diet. In this current study, the nutrient intake did not differ for ewes, although the COP chemical composition revealed a greater content of fiber, which might enhance the generation of propionate in the rumen, which in turn is converted to glucose in the liver, leading to an increased synthesis of lactose in the mammary gland [25]. The increase in milk lactose may also be related to differences in carbohydrate fermentation and glucose supply; however, rumen fermentation and glucose metabolism were not measured in the present study. Therefore, this explanation should be regarded as a possible mechanism rather than a demonstrated effect. Moreover, the numerically higher yield of milk noticed in this study and the previous one might be linked to the COP additional supply of soluble carbohydrates as well as its mild tannin and phenol content that likely enhanced the passage of the feed’s protein to the animals’ duodenum and thereby enhancing its utilization [26]. Hence, our results revealed that COP can be added to ewes’ daily DM intake in amounts ranging from 100 to 200 g/kg of their total mix ration without adversely affecting milk production.

Nutrient digestibility and N balance were not altered by COP inclusion. Nevertheless, the inclusion of coffee pulp in ruminant diets did not negatively impact feed digestibility [27]. The amount of lignin in COP can affect whether the DM is digested. Low digestibility of dietary fiber, especially ADF, is also caused by complex lignin–cellulose linkages. This occurs because rumen microbes find it difficult to break down these bonds, which inhibits cellulolytic bacteria. Polyphenolic chemicals have been shown to reduce fiber digestibility by forming lignocellulose complexes and directly inhibiting cellulolytic bacteria; diets containing 160 g/kg coffee pulp had lower ADF digestibility [9]. However, because rumen microbial populations and fermentation characteristics were not measured here, these mechanisms remain hypothetical for the present study. In this study, adding COP at 100 or 200 g/kg on a DM basis had no adverse effect on nutrient digestibility or N balance.

The addition of COP did not affect the blood metabolites investigated in this study, except BUN. Similar to our findings, BUN was increased by the inclusion of COP in sheep diets [8]. Blood urea nitrogen, on the other hand, was reduced by the addition of COP to goats’ ration [18]. The urea content was within the standard levels of urea in a healthy sheep [28]. Consequently, the diets’ protein content may have exceeded the sheep’s requirements, which might affect its utilization [29]. Higher content of phenolic compounds, polyphenols, and caffeine, which are found in COP, might also affect the concentration of BUN [30]. In our study, other tested blood metabolites such as AST and ALT were not altered by the inclusion of COP, which indicates that using COP in ewes’ diets had no adverse effect on their health. The increase in BUN represents a treatment-related change in N metabolism rather than a demonstrated health benefit. Creatinine, rather than AST or ALT, is the more directly relevant biochemical marker among the variables measured for evaluating renal status, while AST and ALT are primarily associated with hepatic or tissue status.

## 5. Conclusions

Under the conditions of this study, inclusion of coffee pulp up to 200 g/kg DM maintained ewe body weight, lamb growth, milk yield, most milk components, nutrient digestibility, and nitrogen balance. Milk lactose increased, while BUN was higher in COP-fed ewes; the other measured serum biochemical variables were not significantly affected. The corrected economic calculations indicated lower estimated milk-production cost with coffee pulp inclusion. These findings support the inclusion of coffee pulp at up to 200 g/kg DM in diets of lactating Awassi ewes under the conditions tested, while further studies are needed to establish effects across breeds, production systems, and higher inclusion levels.

## Figures and Tables

**Table 1:** Ingredients and chemical composition of diets fed to Awassi ewes.

Item	Diet ^1^		
	CON	COP100	COP200
Ingredients (% DM)			
Barley grain	52.0	56.0	60.0
Soybean meal, 44.0% CP (solvent)	21.0	17.0	13.0
Wheat straw	25.0	15.0	5.0
Coffee pulp ^2^	0.0	10.0	20.0
Salt	1.0	1.0	1.0
Limestone	0.9	0.9	0.9
Vitamin–mineral premix ^3^	0.1	0.1	0.1
Feed cost/ton (US$) ^4^	426	380	334
Nutrients ^5^			
Dry matter, % DM	91.3	90.5	90.2
Crude protein, % DM	15.8	15.9	16.0
Neutral detergent fiber, % DM	30.9	29.7	28.4
Acid detergent fiber, % DM	14.1	13.8	13.6

^1^ The diets were the control diet (CON), 100 g/kg coffee pulp (COP100), and 200 g/kg COP (COP200) of dietary dry matter (DM). ^2^ Contained 168 g/kg CP, 517 g/kg NDF, and 339 g/kg ADF DM basis. ^3^ Composition per kg contained: vitamin A, 600,000 IU; vitamin D3, 200,000 IU; vitamin E, 75 mg; vitamin K3, 200 mg; vitamin B1, 100 mg; vitamin B5, 500 mg; lysine, 0.5%; DL-methionine, 0.15%; manganese oxide, 4000 mg; ferrous sulphate, 15,000 mg; zinc oxide, 7000 mg; magnesium oxide, 4000 mg; potassium iodide, 80 mg; sodium selenite, 150 mg; copper sulphate, 100 mg; cobalt phosphate, 50 mg; dicalcium phosphate, 10,000 mg. ^4^ Feed cost was calculated from the inclusion rate and purchase price of each dietary ingredient in 2026. The calculated costs were US$426, US$380, and US$334 per ton for CON, COP100, and COP200, respectively. ^5^ Dietary DM, CP, NDF, and ADF values are the analyzed values.

**Table 2:** Effects of feeding coffee pulp (COP) on the nutrient intake of Awassi ewes fed lactation diets.

Item	Diet ^1^				
	CON (*n* = 10)	COP100 (*n* = 10)	COP200 (*n* = 10)	SEM	*p* Value
Nutrient intake					
Dry matter, g/d	1744	1726	1715	67.1	0.9055
Crude protein, g/d	276	275	274	9.9	0.3009
Neutral detergent fiber, g/d	539	510	487	53.7	0.6354
Acid detergent fiber, g/d	246	238	232	15.0	0.6659

^1^ The diets were the control diet (CON), 100 g/kg coffee pulp (COP100), and 200 g/kg COP (COP200) of dietary dry matter (DM).

**Table 3:** Effect of feeding coffee pulp (COP) on the body weight of ewes and the growth rate of lambs.

	Diets ^1^				
Item	CON (*n* = 10)	COP100 (*n* = 10)	COP200 (*n* = 10)	SEM	*p* Value
Ewes					
Initial body weight (kg)	58.8	58.7	57.3	2.80	0.837
Final body weight (kg)	55.5	56.2	55.0	2.45	0.886
Body weight change (kg)	−3.3	−2.5	−2.3	0.79	0.422
Lambs					
Initial body weight (kg)	4.9	4.7	4.9	0.16	0.299
Final body weight (kg)	19.2	19.2	19.9	0.50	0.264
Total gain (kg)	14.2	14.5	15.0	0.49	0.290
Average daily gain (g)	254.3	258.8	268.2	8.68	0.290

^1^ The diets were the control diet (CON), 100 g/kg coffee pulp (COP100), and 200 g/kg COP (COP200) of dietary dry matter (DM).

**Table 4:** Effect of feeding coffee pulp (COP) on milk yield and composition of Awassi ewes fed lactation diets.

	Diets ^1^				
Item	CON (*n* = 10)	COP100 (*n* = 10)	COP200 (*n* = 10)	SEM	*p* Value
Milk yield (g/d)	1940.0	2018.7	2037.3	97.35	0.757
Milk composition (%)					
Total Solids	19.64	19.31	19.33	0.221	0.504
Solid-not-fat	11.59	11.50	11.41	0.087	0.372
Protein	6.40	6.25	6.28	0.050	0.106
Fat	8.05	7.81	7.92	0.206	0.720
Lactose	4.56 ^a^	4.73 ^b^	4.69 ^b^	0.039	0.015
Milk composition yield (g/d)					
Total Solids	380.48	390.94	392.72	19.003	0.887
Solid-not-fat	224.79	231.65	232.21	10.753	0.887
Protein	124.21	126.85	128.27	6.741	0.911
Fat	155.69	159.29	160.50	8.950	0.925
Lactose	88.31	95.50	95.37	4.520	0.447
MEV, kcal/kg ^2^	311.35	308.43	309.51	1.828	0.528
ECM, kg/d ^3^	3.51	3.60	3.63	0.188	0.895
Feed conversion ratio (FCR) ^4^	0.91	0.89	0.86	0.064	0.703
Cost/kg milk production (US$) ^5^	0.359 ^c^	0.325 ^b^	0.300 ^a^	2.430	0.002

^1^ The diets were the control diet (CON), 100 g/kg coffee pulp (COP100), and 200 g/kg COP (COP200) of dietary dry matter (DM). ^2^ MEV: milk energy value. ^3^ ECM: energy-corrected milk. ^4^ FCR was calculated as DM intake divided by milk yield. ^5^ Milk-production cost was calculated as: daily DM intake (kg/d) × diet cost (US$/kg) ÷ milk yield (kg/d). ^a–c^ Row means that do not have the same letter differ, *p* < 0.05.

**Table 5:** Effect of feeding coffee pulp (COP) on nutrient digestibility and nitrogen (N) balance of Awassi ewes fed lactation diets.

	Diets ^1^				
Item	CON (*n* = 5)	COP100 (*n* = 5)	COP200 (*n* = 5)	SEM	*p* Value
Digestibility, %					
Dry matter	88.10	87.12	85.87	0.909	0.105
Crude protein	82.79	84.27	80.38	2.12	0.240
Neutral detergent fiber	75.30	73.94	72.83	1.361	0.251
Acid detergent fiber	71.36	72.43	69.21	2.45	0.445
N Balance					
N intake, g/d ^2^	68.04	68.30	67.25	0.697	0.335
N feces, g/d	11.73	10.92	13.18	1.079	0.168
N urine, g/d	9.81	10.24	9.00	0.847	0.376
N retained, g/d	46.53	47.14	45.07	1.057	0.193
N retention, g/100 g	68.36	69.04	67.06	1.625	0.497

^1^ The diets were the control diet (CON), 100 g/kg coffee pulp (COP100), and 200 g/kg COP (COP200) of dietary dry matter (DM). ^2^ The N intake values were obtained from the separate metabolic trial involving 5 ewes per treatment. Therefore, they were different from the N intake values reported for the full experimental population in Table 2.

**Table 6:** Effects of feeding coffee pulp (COP) on the blood metabolites of Awassi ewes fed lactation diets.

Item ^2^	Diet ^1^				
	CON (*n* = 10)	COP100 (*n* = 10)	COP200 (*n* = 10)	SEM	*p* Value
Blood urea nitrogen, mg/dL	19.96 ^a^	23.37 ^b^	24.91 ^b^	1.158	0.017
Glucose, mg/dL	55.80	53.20	55.45	2.987	0.802
Cholesterol, mg/dL	44.70	46.60	47.92	3.031	0.754
HDL, mg/dL ^2^	26.15	28.50	26.60	1.447	0.485
LDL, mg/dL ^2^	16.08	17.04	18.64	2.545	0.103
Triglycerides, mg/dL	12.35	15.62	14.59	1.500	0.305
Creatinine, mg/dL	0.73	0.72	0.71	0.069	0.973
AST, IU/L ^2^	51.90	43.50	41.45	4.290	0.208
ALT, IU/L ^2^	5.10	4.10	4.73	0.362	0.162
ALP, IU/L ^2^	49.35	51.28	50.12	4.908	0.962

^1^ The diets were the control diet (CON), 100 g/kg coffee pulp (COP100), and 200 g/kg COP (COP200) of dietary dry matter (DM). ^2^ HDL: high-density lipoprotein; LDL: low-density lipoprotein; AST: aspartate aminotransferase; ALT: alanine aminotransferase; ALK-P: alkaline phosphatase. ^a,b^ Row means that do not have the same letter differ, *p* < 0.05.

## Data Availability

The original contributions presented in this study are included in the article. Further inquiries can be directed to the corresponding author.
